# Rapid and Sensitive Detection of Novel Avian-Origin Influenza A (H7N9) Virus by Reverse Transcription Loop-Mediated Isothermal Amplification Combined with a Lateral-Flow Device

**DOI:** 10.1371/journal.pone.0069941

**Published:** 2013-08-01

**Authors:** Yiyue Ge, Bin Wu, Xian Qi, Kangchen Zhao, Xiling Guo, Yefei Zhu, Yuhua Qi, Zhiyang Shi, Minghao Zhou, Hua Wang, Lunbiao Cui

**Affiliations:** 1 Institute of Pathogenic Microbiology, Jiangsu Provincial Center for Disease Control and Prevention, Key Laboratories of Enteric Pathogenic Microbiology, Ministry of Health, Nanjing, China; 2 Department of Acute Infectious Diseases Control and Prevention, Jiangsu Provincial Center for Disease Prevention and Control, Nanjing, China; University of Texas Medical Branch, United States of America

## Abstract

A severe disease in humans caused by a novel avian-origin influenza A (H7N9) virus emerged in China recently, which has caused at least 128 cases and 26 deaths. Rapid detection of the novel H7N9 virus is urgently needed to differentiate the disease from other infections, and to facilitate infection control as well as epidemiologic investigations. In this study, a reverse transcription loop-mediated isothermal amplification combined with a lateral flow device (RT-LAMP-LFD) assay to rapidly detect H7N9 virus was developed and evaluated. The RT-LAMP primers were designed to target the haemagglutinin (HA) and neuraminidase (NA) genes of H7N9 virus. Results of 10-fold dilution series assays showed that analysis of RT-LAMP products by the LFD method was as sensitive as real-time turbidity detection, and that the analytic sensitivities of the HA and NA RT-LAMP assays were both 10 copies of synthetic RNA. Furthermore, both the assays showed 100% clinical specificity for identification of H7N9 virus. The performance characteristics of the RT-LAMP-LFD assay were evaluated with 80 clinical specimens collected from suspected H7N9 patients. The NA RT-LAMP-LFD assay was more sensitive than real time RT-PCR assay. Compared with a combination of virus culture and real-time RT-PCR, the sensitivity, specificity, positive predictive value, and negative predictive value of the RT-LAMP-LFD assay were all 100%. Overall, The RT-LAMP-LFD assay established in this study can be used as a reliable method for early diagnosis of the avian-origin influenza A (H7N9) virus infection.

## Introduction

Since February, 2013, a previously unrecognized novel avian-origin influenza A (H7N9) virus associated with human deaths has emerged in China [1]. Although the transmission of avian influenza virus subtypes H5, H7, and H9 to human has been reported early [2], it is the first time that N9 subtype influenza virus has infected human beings. Phylogenetic analysis has shown that all the genes of the novel H7N9 viruses are of avian origin, and are recombined from at least three influenza virus lineages [1], [3]. Compared with avian influenza A (H5N1) virus, it seems to be easier for the novel H7N9 virus to transmit from animals to human. As of 2 May 2013, there have been 128 laboratory-confirmed cases of human infection with H7N9 virus including 26 fatalities [4]. Although different strains of the novel H7N9 virus have been isolated from poultries, birds and the environmental specimens (Data from Global Initiative on Sharing All Influenza Data, GISAID), the source of infection in most of the human cases still remains to be determined. So far, there has been no direct evidence of human-to-human transmission of this virus, however, the presence of mutations in the polymerase basic protein 2 (PB2) gene associated with improved replication of avian influenza viruses in mammals might indicate a certain propensity of the H7N9 virus to further adapt to humans [1], [3]. Therefore, the potential for the novel influenza A (H7N9) virus to spread among the human population is still exist.

A rapid and sensitive methodology for the diagnosis of H7N9 infection is urgently needed to facilitate clinical care, infection control, and epidemiologic investigations. Methods based on PCR are more rapid and sensitive than traditional techniques including virus isolation and serological assays. Real-time RT-PCR is at present the powerful molecular diagnostic method for the novel influenza A (H7N9) virus infection [5], [6], however, it requires expensive real-time PCR equipment and highly skilled technicians, which make this method not suitable for use in primary clinical settings or for field use. The Loop-mediated isothermal amplification (LAMP) method allows amplification of DNA with high specificity and sensitivity at a constant temperature of 60–65°C [7]. As the reaction is conducted under isothermal conditions, it can be carried out with a simple water bath so that a thermal cycler is not required. LAMP can also be used to detect RNA template by the use of reverse transcriptase together with DNA polymerase [7], [8]. To date, RT-LAMP methods have been developed to detect various RNA viruses [9]–[12].

LAMP products can be detected by agarose gel electrophoresis, by the use of spectrophotometric equipment to measure turbidity [13], or by visual inspection of turbidity or color changes [14], [15]. As a result, non-specific amplification products may cause false positive results. To help overcome this problem, LAMP products can be identified by restriction endonuclease digestion [7] or by hybridization with specific probes [16]. To further simplify and speed up the process of the LAMP assay, amplicon detection by lateral flow device (LFD) was successfully applied [17]. In this study, a RT-LAMP amplification combined with a LFD (RT-LAMP-LFD) assay was developed for the rapid detection of novel avian-origin influenza A (H7N9) virus. The characteristics of simplicity (without the need for complex or expensive equipment), rapidity (results can be obtained within 1****h), and excellent sensitivity and specificity make this RT-LAMP-LFD method more suitable for use in low-equipment setting laboratory.

## Materials and Methods

### Ethics statement

Written informed consent for the use of the clinical specimens was obtained from all patients involved in this study. This study was approved by the Ethics Committee of the Jiangsu Provincial Center for Disease Prevention and Control.

### Viral isolates and clinical specimens

H7N9 virus isolates were cultured in Madin-Darby canine kidney (MDCK) cells using standard techniques. An isolated A/Nanjing/1/2013 (H7N9) virus was used as the reference virus. Other genetically and clinically related virus isolates including seasonal influenza viruses (A/H1N1, A/H3N2, and B), 2009 swine-origin influenza virus A/H1N1, avian influenza viruses (A/H5N1 and A/H9N1), parainfluenza viruses (types 1, 2, 3, and 4), human coronaviruses (229E, OC43, HKU1, and NL63) and respiratory syncytial viruses (types A and B) were used as control viruses to assess the specificity of the RT-LAMP assay. A total of 80 respiratory clinical specimens (65 pharyngeal swabs, 7 sputa, and 8 tracheal aspirates) were collected from suspected H7N9 patients in the acute phase of illness. All these isolates and specimens were stored at −80°C until use.

### Extraction of viral RNA

The genomic viral RNA was extracted from 200 μl of samples by using a High Pure viral RNA kit (Roche Diagnostics, Manheim, Germany) according to the manufacturer's instructions. The RNA was eluted in a final volume of 100 μl of elution buffer and kept at −80°C until further analysis.

### Preparation of virus RNA standards

The haemagglutinin (HA) and neuraminidase (NA) genes were amplified from the H7N9 virus strain A/Nanjing/1/2013 (H7N9) with primers containing T7 promoter sequence in the reverse sides (**Table 1**) and were *in vitro* transcribed with T7 RNA polymerase (TaKaRa Biotechnology Co. Ltd., Dalian, China) according to the manufacturer's instructions. The synthetic RNA transcripts were then purified, quantified, mixed in equal-molar amounts, and ten-fold diluted ranging from 10^7^ to10^1^ RNA copies/μl.

**Table 1:** Primers used for *in vitro* transcription of virus RNA standards.

Name	Sequence (5′–3′)	Position
HA-Forward	CCTGGTATTCGCTCTGATTGC	15–35
HA-Reverse	**ACTCGTTAATACGACTCACTATAGGGAG** aGCACCGCATGTTTCCATTCT	1668–1649
NA-Forward	TCTATGCACTTCAGCCACTG	21–40
NA-Reverse	**ACTCGTTAATACGACTCACTATAGGGAG** aCCATCAGGCCAGTTCCATTG	1373–1354

a T7 RNA polymerase promoter sequence.

### Primer design for RT-LAMP

**Figure 1:**
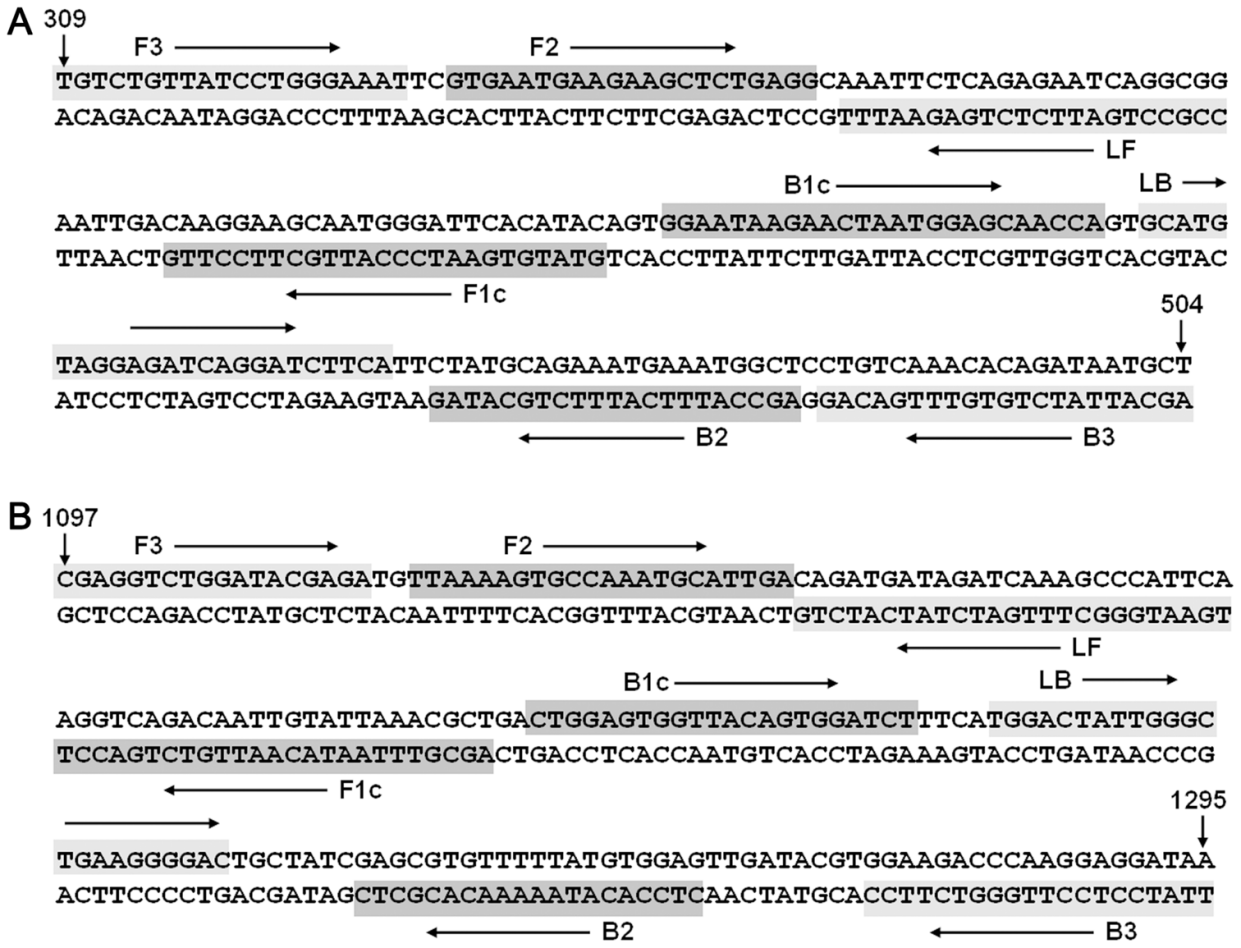
Schematic showing location of RT-LAMP primer binding sites within HA and NA genes. (A) HA RT-LAMP primer binding sites. Assay spans region from nucleotide 309–504 with reference to the HA gene sequence of the H7N9 virus strain A/Nanjing/1/2013 (H7N9). (B) NA RT-LAMP primer binding sites. Assay spans region from nucleotide 1097–1295 with reference to the NA gene sequence of the H7N9 virus strain A/Nanjing/1/2013 (H7N9).

Two separate RT-LAMP assays (six primers each) were designed according to HA and NA gene sequences of the reference virus using a PrimerExplorer V4 program (http://primerexplorer.jp/e/). The feasibility and specificity of the primers were subsequently checked by BLAST search against published genome sequences of the novel H7N9 viruses from the GISAID EpiFlu database and against NCBI database. Details of the final primers are shown in **Figure 1** and **Table 2**. All the primers were synthesized by Sangon (Shanghai, China).

**Table 2:** Details of primers used for RT-LAMP assay.

Target	Name	Type	Position	Sequence (5′–3′)
HA	F3	Forward outer	309–328	TGTCTGTTATCCTGGGAAAT
	B3	Reverse outer	504–484	AGCATTATCTGTGTTTGACAG
	FIP (F1c+F2)	Forward inner	F1c: 405–381	GTATGTGAATCCCATTGCTTCCTTG
			F2: 331–351	GTGAATGAAGAAGCTCTGAGG
	BIP (B1c+B2)	Reverse inner	B1c: 409–433	GGAATAAGAACTAATGGARCAACCA
			B2: 482–462	AGCCATTTCATTTCTGCATAG
	LF a	Forward loop	374–353	CCGCCTGATTCTCTGAGWATTT
	LB b	Reverse loop	436–459	GCATGTAGGAGATCAGGATCTTCA
NA	F3	Forward outer	1097–1114	CGAGGTCTGGATACGAGA
	B3	Reverse outer	1295–1276	TTATCYTCCTTGGGTCTTCC
	FIP (F1c+F2)	Forward inner	F1c: 1188–1164	AGCGTTTARTACAATTGTCTGACCT
			F2: 1117–1138	TTAAAAGTGCCAAATGCATTGA
	BIP (B1c+B2)	Reverse inner	B1c: 1191–1212	CTGGAGTGGTTACAGTGGATCT
			B2: 1266–1247	YTCCACATAAAAACACGCTC
	LF a	Forward loop	1163–1139	TGAATGGGCTTTGATCTATCATCTG
	LB b	Reverse loop	1217–1239	TGGACTATTGGGCTGARGGGGAC

a 5′-Labeled with FITC when used in RT-LAMP-LFD assay.

b 5′-Labeled with biotin when used in RT-LAMP-LFD assay.

### RT-LAMP reaction and product detection

RT-LAMP was carried out with a RNA Amplification Kit (RT-LAMP) (Eiken China Co., Ltd., Shanghai, China) in a final volume of 25 µl containing 0.2 µM each of the outer primers F3 and B3, 2.0 µM each of the primers FIP and BIP, and 1.0 µM each of the primers LF and LB (labeled or not). The reaction mixture containing distilled water was used as negative controls. RT-LAMP amplification reactions were carried out at 60, 63, and 65°C for 60 min using a real-time turbidimeter (LA320C, Teramecs, Tokyo, Japan). The RT-LAMP products were also detected by a LFD (Ustar Biotech Co., Ltd., Hangzhou, China) as described previously [17]. The principle is primers LF and LB are conjugated to fluorescein isothiocyanate (FITC) and biotin, respectively, during a positive RT-LAMP reaction both tags are incorporated in the amplicons resulting in a visible test line. The LFD detection also utilizes a control line to indicate whether the unit has functioned correctly.

### Sensitivity of RT-LAMP by real-time turbidity detection and LFD

To compare test sensitivity, RNA was extracted from the culture supernatants of H7N9 virus strain A/Nanjing/1/2013 (H7N9)-infected MDCK cells. Ten-fold serial dilutions of RNA were used as templates for RT-LAMP reactions at 63°C for 1 h. Besides real-time turbidity detection, The RT-LAMP products were simultaneously detected by LFD as described above.

### Analytical sensitivity and specificity of the RT-LAMP-LFD

Ten-fold serial dilutions of synthetic H7N9 viral RNA transcripts of HA and NA genes (ranging from 10^7^ to10^1^ RNA copies) were used to assess the detection limits of the HA and NA RT-LAMP-LFD assays. The specificities of the RT-LAMP-LFD assays were determined by analyzing the RNA extracts from various control viruses mentioned above and the reference virus.

### Real-time RT-PCR detection of H7N9 virus

Real-time RT-PCR for detection of H7N9 virus was carried out using the primers and probes recommended by the WHO Collaborating Centre in Beijing [5] and the SuperScript III Platinum One-Step qRT-PCR Kit (Invitrogen) according to the instructions. The cycling conditions were composed of 20 min at 50°C, 2 min at 95°C, followed by 40 cycles with 94°C for 15 s and 60°C for 1 min, and a final extension cycle of 72°C for 10 min.

### Evaluation of the RT-LAMP-LFD assay with clinical specimens

To evaluate the performance characteristics of the RT-LAMP-LFD assay, a reference standard which represents the combined results of the viral culture and real-time RT-PCR was established. A sample was determined to be positive when either viral culture or real-time RT-PCR was positive. Totally 65 pharyngeal swabs, 7 sputa, and 8 tracheal aspirates with suspicious H7N9 infection were extracted as described above and analyzed by RT-LAMP-LFD assay and the reference methods.

## Results

### Optimization of RT-LAMP reaction temperature

The real-time turbidimetry device enables observation of primer kinetics. At the commencement of the study, we used a real-time turbidimeter to determine the optimal RT-LAMP reaction temperature for both the HA and NA primer sets. RT-LAMP reactions were carried out for 60 min at 60, 63, and 65°C using diluted RNA extracted from the culture supernatants of H7N9 virus strain. Results showed that the 63°C temperature was the optimal reaction temperature for both HA and NA primer sets (data not shown).

### Comparison of sensitivity between RT-LAMP-turbidity detection and RT-LAMP-LFD

**Figure 2:**
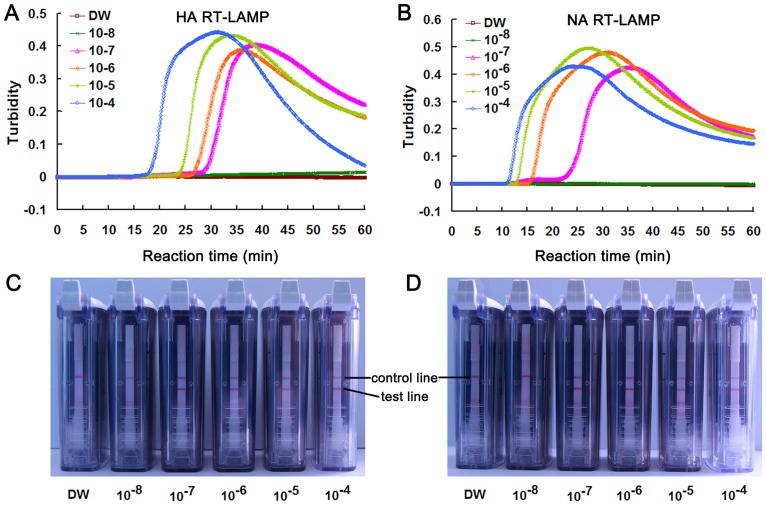
Comparison of real-time turbidity measurement and LFD for the detection of RT-LAMP products. HA (A and C) and NA (B and D) RT-LAMP on 10-fold serial dilutions of H7N9 virus RNA. (A and B) detection using the real-time turbidimetry device. DW: distilled water used as non-RNA negative control. (C and D) detection using LFD. Loop primers were tagged with Biotin and FITC.

Sensitivities of RT-LAMP-turbidity detection and RT-LAMP-LFD were compared using ten-fold serially diluted RNA. To exclude the possible inhibition of RT-LAMP reactions by labeled primers, unlabeled LF and LB primers were used in RT-LAMP monitored by real-time turbidity detection. Analysis of RT-LAMP products by the LFD showed that, the detection limits for HA and NA genes were both 10^−7^ dilutions, which were equivalent to their detection limits obtained by RT-LAMP-turbidity detection. Thus, the LFD method which can provide a result within 5–10 min after completion of the RT-LAMP reaction was as sensitive as real-time turbidity detection (**Figure 2**).

### Analytical sensitivity and specificity of the RT-LAMP-LFD

**Figure 3:**
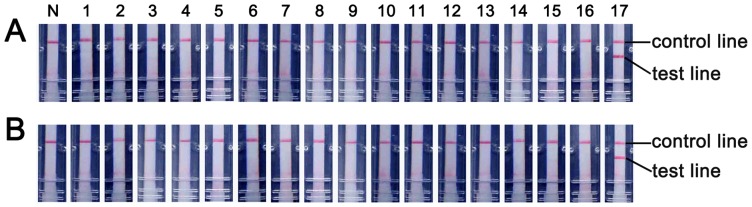
Specificity test results of RT-LAMP-LFD for H7N9 virus detection. The specificities of HA (A) and NA (B) RT-LAMP-LFD assays were determined by analyzing the RNA extracts from various control viruses and the reference virus. N: No template control; 1: human seasonal influenza A H1N1 virus; 2: human seasonal influenza A H3N2 virus; 3: human influenza B virus; 4: 2009 swine-origin influenza virus A H1N1 virus; 5: avian influenza A H5N1 virus; 6: avian influenza A H9N1 virus; 7: parainfluenza virus types 1; 8: parainfluenza virus types 2; 9: parainfluenza virus types 3; 10: parainfluenza virus types 4; 11: human coronavirus 229E; 12: human coronavirus OC43; 13: human coronavirus HKU1; 14: human coronavirus NL63; 15: respiratory syncytial viruses types A; 16: respiratory syncytial viruses types B; 17: H7N9 virus.

The analytical sensitivities of the HA and NA RT-LAMP-LFD assays were assessed using ten-fold serial dilutions of synthetic H7N9 viral RNA transcripts of HA and NA genes (ranging from 10^7^ to10^1^ RNA copies). After 60 min amplification, both the HA and NA RT-LAMP-LFD assays revealed a detection limit of 10 copies of synthetic RNA. To assess the potential for the RT-LAMP-LFD assays to cross-react with other related viruses which could cause similar symptoms, the HA and NA RT-LAMP assays were tested against viral RNA extracts from various control viruses. As shown in **Figure 3**, a positive test line was only observed in the preparation of the reference virus, whereas none of the control viruses showed a positive result in both HA and NA RT-LAMP-LFD assays, indicating the high specificities of both the assays.

### Evaluation of the RT-LAMP-LFD with clinical specimens

To evaluate the performance characteristics of the HA and NA RT-LAMP-LFD assays, a total of 80 clinical specimens collected from suspected H7N9 patients in the acute phase of illness were subjected to RT-LAMP-LFD assay with the parallel analysis by the reference methods. The results showed that, of 80 specimens, 22 were positive for both HA and NA genes as detected by RT-LAMP-LFD, of which 21 were positive for both HA and NA genes as detected by real-time RT-PCR. The HA-positive but NA-negative specimen detected by real-time RT-PCR was further analyzed by viral culture and sequencing, and was verified as H7N9 virus infection, indicating the superior sensitivity of the RT-LAMP-LFD assay. Compared to the reference standard, the sensitivity, specificity, positive predictive value, and negative predictive value of both HA and NA RT-LAMP-LFD assays were all 100% (**Table 3**).

**Table 3:** Performance of RT-LAMP-LFD assay compared with the reference standard for detecting avian-origin influenza A (H7N9) virus.

RT-LAMP-LFD		Reference standard			Performance characteristics (%) a			
		Positive	Negative	Total	Sensitivity	Specificity	PPV	NPV
HA	Positive	22	0	22	100%	100%	100%	100%
	Negative	0	58	58				
	Total	22	58	80				
NA	Positive	22^b^	0	22	100%	100%	100%	100%
	Negative	0	58	58				
	Total	22	58	80				

a PPV, positive predictive value; NPV, negative predictive value.

b One HA-positive while NA-negative sample detected by real-time RT-PCR was further confirmed to be H7N9 infection by viral culture and sequencing.

## Discussion

Major uncertainties still exist with regards to the genetic variability and the pandemic potential of the novel avian-origin influenza A (H7N9) virus which warrant the development of new methods to detect this virus. In this study, we describe a sensitive RT-LAMP-LFD method for the specific detection of H7N9 virus for the first time. The optimal LAMP reaction conditions are 63°C for 60 min, which is more suitable for low-equipment setting laboratory and for on-site testing.

The use of the four specific primers and two loop primers targeting the HA or NA gene of H7N9 virus ensured high specificity of nucleic acid amplification. Both the HA and NA RT-LAMP assays showed 100% specificities for identification of H7N9 virus. Furthermore, loop primers could accelerate the LAMP reaction because they hybridize to the stem-loops, except for those loops that are hybridized by the inner primers and prime strand displacement DNA synthesis [18]. Although 60 min was used for H7N9 RT-LAMP reactions, most of the amplification reactions could be finished within 40 min (**Figure 2**). Thus, the RT-LAMP-LFD assay is faster than real-time RT-PCR.

The concordance of high analytical sensitivity between RT-LAMP and the most sensitive molecular methods for detection of pathogens has been previously reported [19]–[21]. Several possible factors may contribute to this fact. For example, the RT-LAMP reaction was less affected by the presence of various salts, was less sensitive to inhibitors, and was able to tolerate the inhibitory effect of large amounts of templates [22]. In this study, the analytic sensitivities of the HA and NA RT-LAMP assays were both 10 copies of synthetic RNA. Compared to the reference standard, the sensitivity, specificity, positive predictive value, and negative predictive value of the RT-LAMP assays were all 100%.

The RT-LAMP results can be determined by agarose gel electrophoresis, by the use of spectrophotometric equipment to measure turbidity, by naked eye for the presence of a white precipitate derived from magnesium pyrophosphate, or by visualization of the RT-LAMP products under natural light or UV irradiation after adding SYBR green I or calcein dyes. However, due to the use of several primers, RT-LAMP generates a complex mixture of DNA products, and thus these product detection methods cannot distinguish specific and non-specific amplicons. Furthermore, assessment of turbidity or color with the unaided eye is potentially subjective, and there is always the possibility that a sample may be somewhat ambiguous to the naked eye when the concentration of virus is low [23]. Additionally, some dyes such as SYBR green I have adverse effect on LAMP amplification reaction. Gel electrophoresis has been found to be approximately 10×more sensitive than the SYBR green/naked eye inspection [24], nevertheless, electrophoresis after amplification increases the opportunity for product contamination. In the present study, we used a simple LFD utilizing a lateral flow strip housed in an enclosed, sealed plastic device to prevent the leakage of amplicons to objectively detect RT-LAMP products in approximately 5 min [17], [25], [26]. The use of LFD to detect RT-LAMP products not only makes the assay more specific, but also negates the need for electrophoresis equipment and DNA detection equipment. Our results showed that the LFD method was as sensitive as real-time turbidity detection. In some studies, only one labeled primer was used in LAMP reaction, another labeled probe was added into the LAMP amplicons to form double-labeled detectable products [27]–[29]. This would increase the chance of product contamination. In our study, both loop primers were labeled with tags such as FITC and biotin. The results obtained from this study and others [24], [30], [31] demonstrated that the usage of two labeled primers has no adverse effect on LAMP reaction.

In conclusion, these data show that a reliable RT-LAMP-LFD assay has been developed for the detection of novel avian-origin influenza A (H7N9) virus causing the current outbreak, which would facilitate the clinical care, infection control, as well as epidemiologic investigations. The RT-LAMP-LFD assay is specific and sensitive, and does not require expensive equipment. The use of the LFD provides a rapid and objective readout of the assay's results and avoids cross-contamination. This RT-LAMP-LFD assay is especially useful in resource-limited situations such as primary care facilities.
